# Physicochemical Stability of Insulin and Analogues in Saline Infusion: Screening for Amyloid and Amorphous High-Molecular-Weight Material

**DOI:** 10.1021/acsomega.5c12466

**Published:** 2026-03-06

**Authors:** João Gabriel da Cruz e Silva, Fernando de Sá Ribeiro, Luís Maurício T. R. Lima

**Affiliations:** Laboratório de Biotecnologia Farmacêutica - pbiotech, Faculdade de Farmácia, 28125 Universidade Federal do Rio de Janeiro, Rio de Janeiro, RJ 21941-902, Brazil

## Abstract

Insulin is known to form subvisible particles and amyloid material, which can lead to iatrogenic amyloidosis and reduced potency necessary for glycemic control. In the intensive care unit, insulin is commonly diluted in saline (1-unit/mL) for intravenous infusion. While reports of insulin aggregation in such dilutions exist, it remains unclear whether these conditions favor the formation of amyloid or subvisible particles. Here, we report standardized assays for detecting amorphous and amyloid insulin aggregation and applied them to investigate these particles in insulin infusion setups. Amorphous insulin was produced by heating insulin (100 U/mL) in plastic tubes, while amyloid insulin formed within 3 days by incubating in saline or sodium phosphate buffer, pH 7.0. Electron microscopy confirmed the amyloid nature of these aggregates, and dynamic light scattering detected subvisible particles as low as 0.05 U/mL. Insulin incubated at 1-unit/mL in saline showed no detectable amyloid material or subvisible particles up to 48 h at room temperature. These results suggest that insulin diluted to 1-unit/mL in saline does not form detectable amyloid or subvisible particles under the tested conditions and that these analytical approaches may be helpful for other biopharmaceuticals.

## Graphical abstract

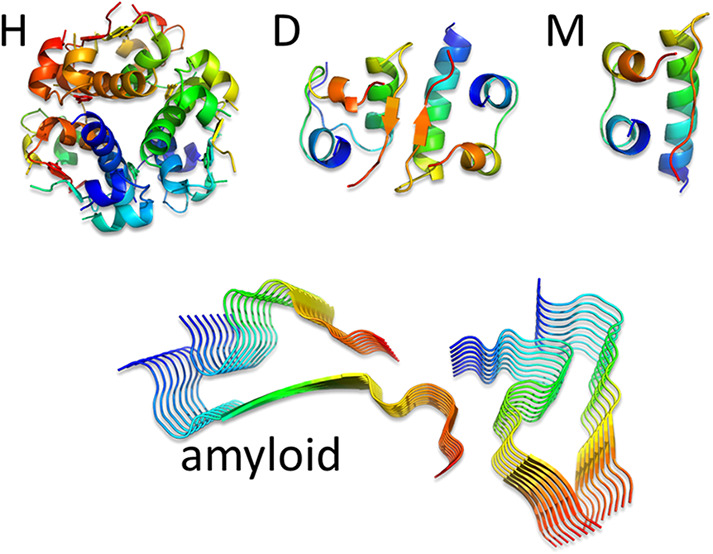

## 1 Introduction

Insulin is a key therapeutic protein for both diabetic individuals and hospital use in patients in the intensive care unit (ICU) requiring tight glycemic control. Insulin formulations are commonly found at 100-unit/mL strength, which may require diluting to 1-unit/mL for continuous infusion, which is known as the insulin infusion protocol (IIP).^1–4^ Typical insulin dilution is performed in saline (0.9% sodium chloride, Ringer’s or lactate Ringer’s injection, or 5% dextrose), by injecting insulin into the diluent flasks or by mixing both in glass or plastic containers. Insulin adsorption onto the container surface (either polymers or glass) and IV tubing may occur, as well as aggregation. Wild-type human insulin (also known as regular insulin) dilution in saline in polyvinyl chloride flasks may result in decrease in concentration from 30 to 60% when refrigerated,^5^ while aspart insulin (^B29^Asp-human insulin) diluted to 1-unit/mL in saline was shown to be stable (better than 90%) at room temperature (25 ± 2 °C) for about one (1) month when stored in polypropylene (PP) syringes or three (3) months when stored in cyclic olefin copolymer (COC) vials.^6^

While insulin quality is highly standardized internationally among pharmacopeias, advances have been made in the identification of physicochemical stability. Insulin has been known for almost a century to form subvisible particles,^7–15^ including the *du Vigneaud* method of producing highly organized amyloid insulin by heating acidic solution,^9^ solved by cryoelectron microscopy (cryoEM).^15^ Human insulin at an original 100-unit/mL strength was found stable for over 28 days in plastic syringes, both refrigerated and at room temperature.^16^ However, human insulin and analogues, such as aspart (^B29^Asp-human insulin) and LisPro (^B28^Lys-^B29^Pro-human insulin), were found to form nonamyloid particles below the subvisible range (2 to 100 μm, according to United States Pharmacopeia (USP), General Chapter <1787>) and high-molecular-weight protein (HMWP; USP <121.1>) in their original vial after first use within 30 days of storage at both 4 and 37 °C,^17^ as detected by dynamic light scattering (DLS).^18^

Despite advances in characterizing the chemical and physical stability of insulin and formulation components over time, it is still unknown whether insulin could form aggregated subvisible particles, either amorphous or amyloid, under saline dilution as used in IIP. Standard methods for amyloid and amorphous aggregates of insulin are also required for the investigation of such material under IIP conditions. In this study, we propose a method for the production of amorphous and amyloid fibrillation assays and the use thereof in the characterization of such HMWP materials in the extemporaneous formulation of insulin in saline for IIP.

## 2 Materials and Methods

### 2.1 Materials

Human insulin (wild-type, regular; Novolin R, leaflet) and analogues (aspart, NovoRapid, leaflet; LisPro, HumaLog, leaflet) were purchased from local drugstores and refrigerated at all times until use. Insulin 100 U/mL corresponds to approximately 3.45 mg/mL. Composition of the insulin products is as described:

- **Human insulin**: zinc 21 μg/mL, glycerol 16 mg/mL, *m*-cresol 3 mg/mL, W.F.I., and HCl/NaOH to pH of approximately 7.0–7.8.
- **LisPro** (^B28^Lys^B29^Pro): 19.7 μg zinc ion, glycerol 16 mg/mL, *m*-cresol 3.15 mg/mL, trace amounts of phenol, Na_2_HPO_4_ 1.0 mg/mL, W.F.I., and HCl/NaOH to pH of approximately 7.0 to 7.8.
- **Aspart** (^B29^Asp): 19.6 μg/mL zinc, 16 mg/mL glycerol, 1.72 mg/mL m-cresol, 1.50 mg/mL phenol, 1.25 mg/mL Na_2_HPO_4_·2H_2_O, 0.58 mg/mL NaCl, W.F.I., and HCl/NaOH to pH of approximately 7.2–7.6.

The assays, in technical replicates, were conducted with insulin within the expiration date and no longer than 30 days after first use. Insulin was withdrawn from the original flask using new sterile syringes from insulin vials as directed by the manufacturer. All other reagents were of analytical grade.

### 2.2 Insulin Amorphous Aggregation and Quantification

Human insulin (100 μL, 100 U/mL; approximately 600 μM) was transferred to a PP tube (nominal capacity 1.5 mL) and boiled for 5 min, followed by centrifugation at 10,000 rpm at room temperature for 5 min. Protein quantification was performed in 96-well plates (transparent, Cralplast, Cat 655111T) by the Bradford method.^19^ The supernatant was transferred to another PP tube, and the remaining precipitate was dispersed with 100 μL of water under vigorous vortexing for 10 s. All samples (intact human insulin, resuspended precipitate, and supernatant) were diluted (20 μL sample + 80 μL water), followed by serial dilution (50 μL sample + 50 μL water) and the addition of 200 μL of the Bradford reagent (Scienco Biotech LTDA). After 15 min incubation at 25 °C covered with a lid and protected from light, the plate was read in a plate reader (Epoch; Biotek Instruments, Inc., USA) at 595 nm. Measurements were accepted only in the linear range; samples with high absorbance were diluted with water to reach the linearity range, and the readings were proportionally corrected for the dilution.

### 2.3 Amyloid Fibrillation Assay

Insulin fibrillation assay was performed in a multiwell plate and monitored by Thioflavin T as reported.^14,20^ Insulin at varying dilutions in saline or 50 mM sodium phosphate buffer (PBS, pH 7.4), in the presence of 30 μM ThT, were incubated in a 96-well, flat-bottom plate (black, Corning, Cat 3915; transparent, Cralplast, Cat 655111T), with or without one glass sphere (3 mm) per well when indicated, and sealed with a transparent film (Crystal Clear Invisible Tape, Duck Brand, USA). Measurements were performed in a fluorescence spectrometer (H1-Synergy plate reader, Biotek, USA; pbiotech, FF, UFRJ), with excitation at 440 nm and emission set at 482 nm, gain 7, top read, with readings each of 5 min preceded by 3 s shaking (490 rpm) at room temperature (25 ± 2 °C).

### 2.4 Dynamic Light Scattering (DLS)

DLS measurements were performed on a DynaPro (Wyatt, USA; LaBiME, FF, UFRJ) at room temperature (25 ± 2 °C) using a 45 μL quartz cuvette (Wyatt; Cat WNQC-45–00). For each sample, 10 accumulations were measured. DLS scattering intensity for control samples after boiling water or saline in similar PP tubes (nominal capacity 1.5 mL) for 5 min results in scattering intensity below 10^3^ counts/s, and no sufficient correlation data were measurable to allow reliable calculation of the hydrodynamic radius (*n* = 3).

### 2.5 Stability Assay in Saline

Sterile saline flasks (0.9% NaCl in water for injection; Farmace, Brazil; Registration MS 1.1085.0001/021-5, ANVISA) were kept at room temperature (25 ± 2 °C) under mild rocking (8 oscillations/min), and aliquots were withdrawn with sterile syringes for immediate measurements in DLS or ThT fluorescence as described above.

### 2.6 Circular Dichroism

Circular dichroism was measured for insulin at 1-U/mL, in a 1.0 mm quartz cuvette at 25 °C (Peltier-controlled) in a spectropolarimeter J-1500 (Jasco Inc., Japan; LTPV, IBqM, UFRJ), with a scan rate of 100 nm/min, 0.2 nm step resolution, and 3 accumulations/spectra. Proper blank subtraction was performed. The spectra were smoothed by using the built-in means-movement method with a convolution width of five points to reduce high-frequency noise while preserving spectral features. Data is shown in the 260–200 nm region corresponding to low HT (Figure S1). Spectra are the means of the data.

### 2.7 Transmission Electron Microscopy

The samples (10 μL) were placed on Parafilm, and Formvar-coated copper grids (300 mesh) were deposited onto the drop for about 5 min and washed with 500–800 μL of cold water. The excess water was drained by capillarity onto a filter paper, and the material was negatively stained by placing the grid onto uranyl acetate 2.0% (5 μL) onto Parafilm for 5 min under the protection of light. Excess uranyl acetate was washed out with cold water (500–800 μL) and the excess drained by capillarity using filter paper. The grids were allowed to dry out in a desiccator for about 1 day. Images were acquired in an HT7800 transmission electron microscope (TEM) (Hitachi; UMA-3, CENABIO, UFRJ) operating at 100 kV.

## 3 Results

### 3.1 Detection Limit of High-Molecular-Weight Insulin by DLS

Standardization of amorphous aggregated insulin was obtained by heating. Human insulin was subjected to heat (boiling water) in a polypropylene (PP) tube for 5 min. Turbidity was promptly observed. The heated samples were centrifuged, and the amounts of insulin in the precipitate and supernatant were quantified. The precipitate material obtained after boiling and centrifugation represented most of the total protein according to quantification (Figure 1).

**Figure 1:**
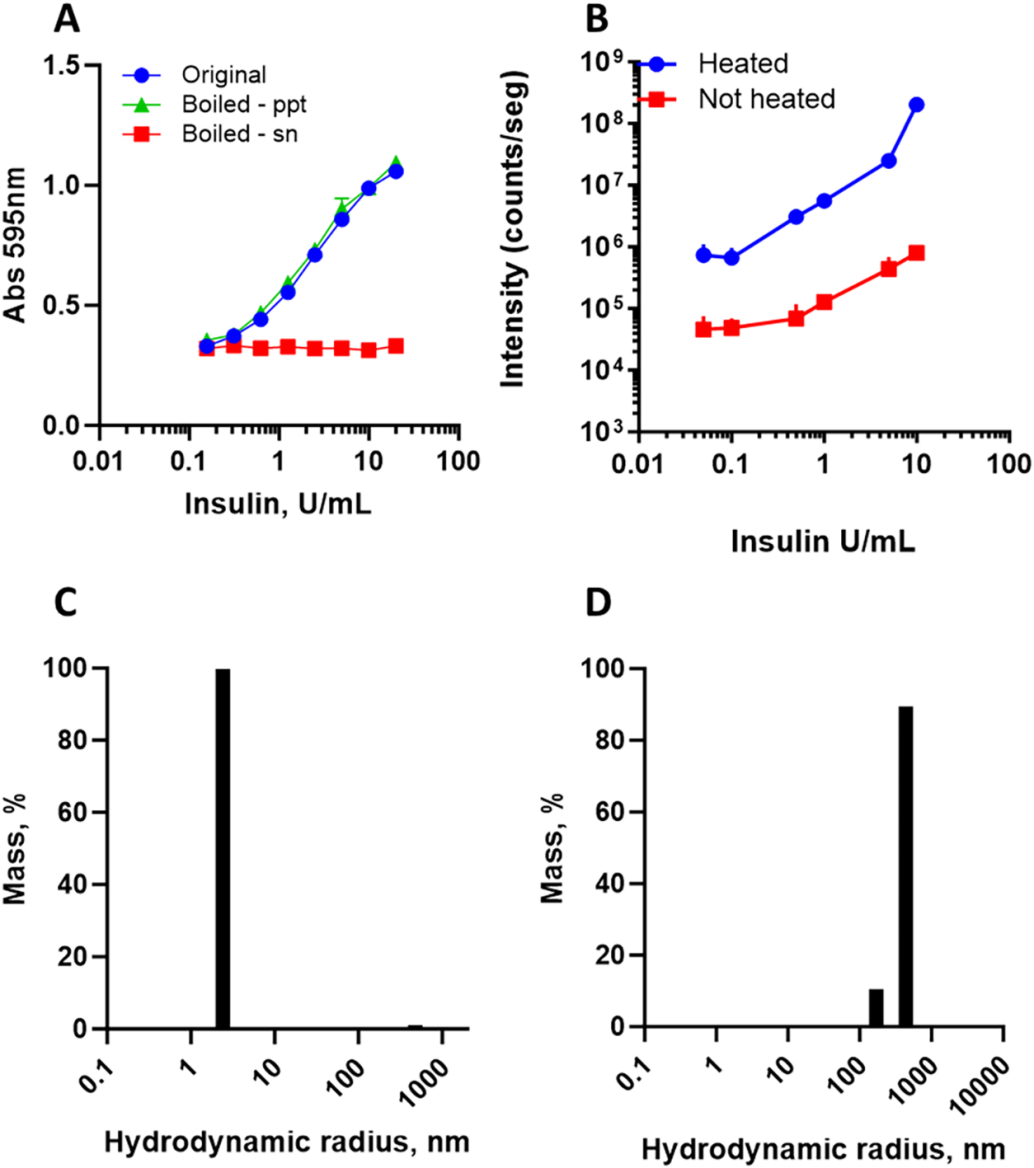
Standardization of amorphous aggregated insulin. (A) Insulin was subjected to heat (boiling water) for 5 min, centrifuged for 5 min at room temperature, and the amount of insulin in the precipitate (suspended in water for the same original volume) and supernatant was serially diluted with water, quantified, and compared to the original insulin not heated. Symbols are average and standard deviation. Curves converge to A_595 nm_ of about 0.34, which corresponds to plate plastic and water (0.04) and the Bradford reagent in the absence of protein (0.30). Intact and aggregated (boiled for 5 min) human insulin (100 U/mL) was serially diluted with saline, and DLS was measured. (B) Dependence of total DLS intensity on insulin concentration (*n* = 3; bars are standard deviation). (C) Particle distribution profile for intact insulin (10 IU/mL in saline). (D) Particle distribution profile for aggregated insulin (10 IU/mL in saline).

The insulin samples were analyzed by DLS, showing a higher signal for the aggregated sample (Figure 1B). Serial dilution of the insulin follows a progressive decrease in dynamic light scattering (DLS) intensity in 2 orders of magnitude, which is higher for aggregated insulin compared to native insulin (Figure 1B). Aggregated insulin was detected close to 10^4^-fold dilution (to 0.05-unit/mL), i.e., 5% of the IIP concentration (of 1 IU/mL). The intact insulin shows a particle size distribution at about 2 nm (Figure 1C), while the aggregated insulin presents a particle size distribution in the DLS between 100 and 1000 nm (Figure 1D).

### 3.2 Standardization of Amyloid Aggregation of Insulin

Insulin amyloid fibrillation at 1 IU/mL (34.5 μg/mL) was screened in a 96-well plate in both saline and sodium phosphate buffer, pH 7.4 (PBS), and monitored by Thioflavin T (ThT) fluorescence. Both human insulin and the analogues aspart and LisPro showed an increase in ThT fluorescence after a lag phase, indicative of amyloid fibril formation, both in PBS and saline (Figure 2). The amyloid fibrillation curve follows a typical behavior comprising a lag phase, an exponential phase, and a plateau at about 72 h of incubation. Insulin fibrillation in saline occurs even at low insulin concentration (0.1 IU/mL; 3 μg/mL; Figures S2 and S3).

**Figure 2:**
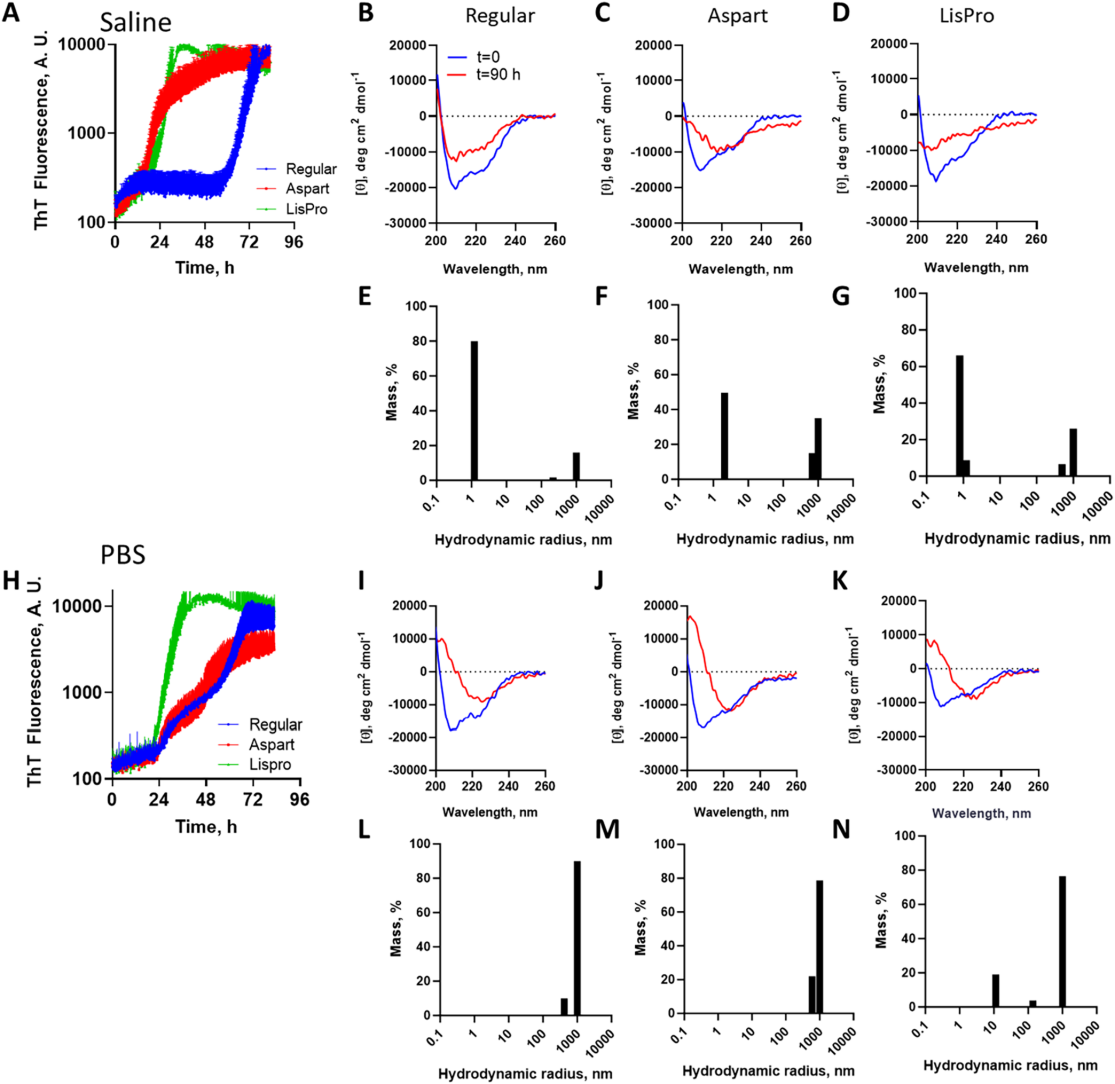
Insulin amyloid aggregation. Insulin (1 IU/mL; regular human, blue; aspart, red; LisPro, green) was incubated in either (A to G) saline or (H to N) 50 mM sodium phosphate buffer pH 7.0, and incubated at 25 °C in a multiwell black plate, in the presence of glass sphere (3 mm), and ThT fluorescence (30 μM) was measured at 5 min time interval with 3 s linear shaking before measurements (*n* = 4; bars are standard deviation). The end-point of measurements performed in parallel in the absence of ThT was evaluated for secondary structure by circular dichroism (baseline in blue, 90 h incubation in red) without further dilution, and compared to insulin immediately diluted in the corresponding assay solution (saline or phosphate) for regular human (B and I), aspart (C and J) and LisPro (D and K) insulin. The end-point was evaluated for the presence of subvisible high-molecular-weight aggregates by DLS for regular human (E and L), aspart (F and M), and LisPro (G and N) insulin.

The human insulin and analogues that underwent amyloid fibrillation were evaluated for changes in their secondary-structure profiles using circular dichroism. Both human insulin and analogues showed minima at about 212 nm and at about 222 nm, corresponding to a typical α-helix, with little difference between saline and phosphate buffer. After 90 h under fibrillation conditions, all insulins showed reduced signal in the far-UV region (Figure 2), indicating decreased secondary-structure content, which might be due to both changes in conformation and loss of signal due to aggregation. Additionally, the spectral profile showed loss of signal in the region of 212 nm and prevalence of negative signal at about 222 nm for all insulins in PBS, and for the aspart analogue in saline, indicative of the interconversion from the α-helix content into β-sheet-rich structures, characteristic of amyloid fibrils. While native insulin shows a small hydrodynamic radius at baseline (human = 2.00 + 0.46 nm; Aspart = 3.03 + 1.78 nm; LisPro = 1.03 + 0.22 nm; *n* = 3; mean and standard deviation of the mean; Table S1), the end-point of the fibrillation assay showed high-molecular-weight subvisible particles, as indicated by DLS (Figure 2).

### 3.3 Morphology of Insulin Aggregates

The end-point of the fibrillation kinetics was evaluated by transmission electron microscopy (TEM; Figure 3). The kinetic fibrillation assays (Figures 2 and 3) were conducted for over 3 days in order to achieve a plateau in the fibrillation assays, since interrupting at 72 h would result in an incomplete fibrillation curve. Kinetics performed at 1-unit/mL insulins (human, aspart, and LisPro) in saline (Figure 3A) and in PBS (Figure 3E) showed amyloid fibrils, confirming the tinctorial identification by ThT. These data provide morphological evidence for amyloid formation by insulin under fibrillation conditions in multiwell plates, which can be achieved in both saline and PBS, and ThT behaves as a key quantitative biomarker.

**Figure 3:**
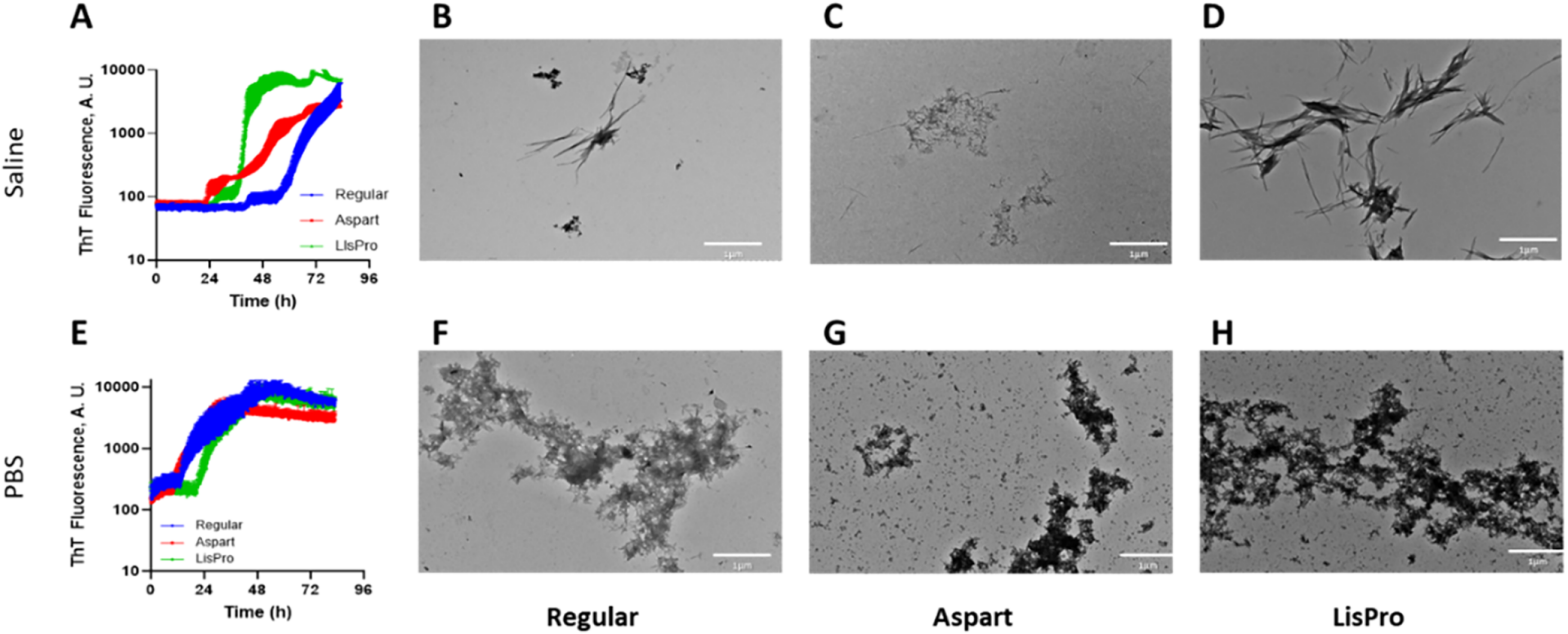
Morphology of aggregation product by transmission electron microscopy (TEM). Insulin (1 IU/mL; regular human, blue; aspart, red; LisPro, green) was incubated in multiwell black plate, at 25 °C in either saline (A to D) or PBS (50 mM sodium phosphate buffer pH 7.0; E to H) with 30 μM ThT (E to H), and ThT fluorescence was measured at 5 min time interval with 3 s linear shaking before measurements (*n* = 4; bars are standard deviation) for 83 h. After the kinetics, aliquots were taken for the evaluation of morphology by TEM as described in the experimental section. (B and F) Regular human insulin; (C and G) Aspart; (D and H), LisPro. Bars = 1 μm.

### 3.4 Screening for Amyloid and Subvisible Particles in Insulin Infusion Setups

While insulin fibrillation can occur in saline and PBS, with differences in kinetics and morphology (Figures 2 and 3), only saline is appropriate for clinical practice. Human insulin dilution in saline at 1 IU/mL (as in IIP) was screened for amyloid by ThT measurements and subvisible particles by DLS. No increase in ThT signal from baseline was observed over 2 days of incubation of insulin in saline, both at quiescent and under mild horizontal oscillation (Figure 4). Evaluation of DLS of insulin in saline flasks revealed only particles compatible with native insulin, with no detectable high-molecular-weight particles. Collectively, these data indicate that under typical conditions for IIP, no amyloid material or subvisible particles are detectable up to 48 h at 25 °C within the analytical limits shown here.

**Figure 4:**
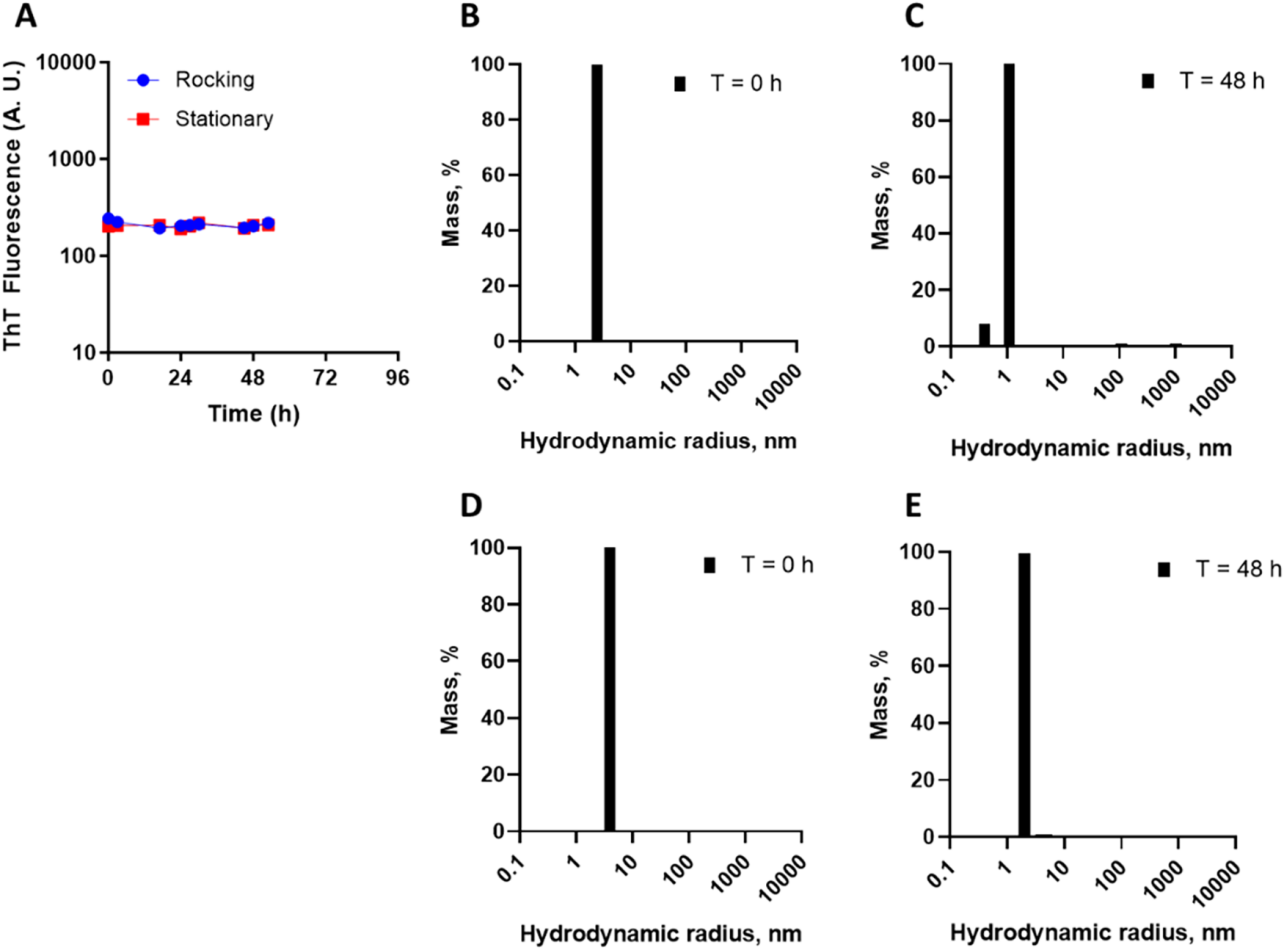
Screening for amyloid in insulin infusion setups by ThT and DLS. Insulin (1 IU/mL) was incubated in saline flasks at 25 °C and aliquoted with sterile syringes for the evaluation of amyloid material by ThT fluorescence under similar instrumental conditions to previous measurements. (A) Evaluation of the effect of mild rocking (8 oscillations/min) on the formation of ThT-responsive material (*n* = 3; bars are standard deviation). DLS was evaluated at 0 h (B and D) and after 48 h (C and E) of incubation, in quiescent (B and C) or mild agitation (D and E) conditions. Symbols are the average and standard deviation of three independent measurements from different saline flasks.

We further evaluated the potential for aggregation of human and analogue insulins (LisPro and Aspart) in a saline flask for IIP. Insulins were diluted in saline flasks, incubated for 72 h at 25 °C, and screened for amyloid formation by ThT, DLS, and CD. No ThT-responsive material was detected, as well as no subvisible particle (by DLS) or secondary-structure changes (by CD) were detected (Figures 5 and S4). The IIP in clinical practice typically recommends changing the infusion every 24 h. We performed the simulated IIP for over 2 days (53 h in Figure 4, and 72 h in Figure 5) in order to understand the behavior of the protein under the IIP conditions beyond the recommended 24 h limit. These data suggest that under conditions similar to those used in the IIP, no amyloid material is detected up to 72 h using these techniques, indicating conformational stability of these insulin products in saline.

**Figure 5:**
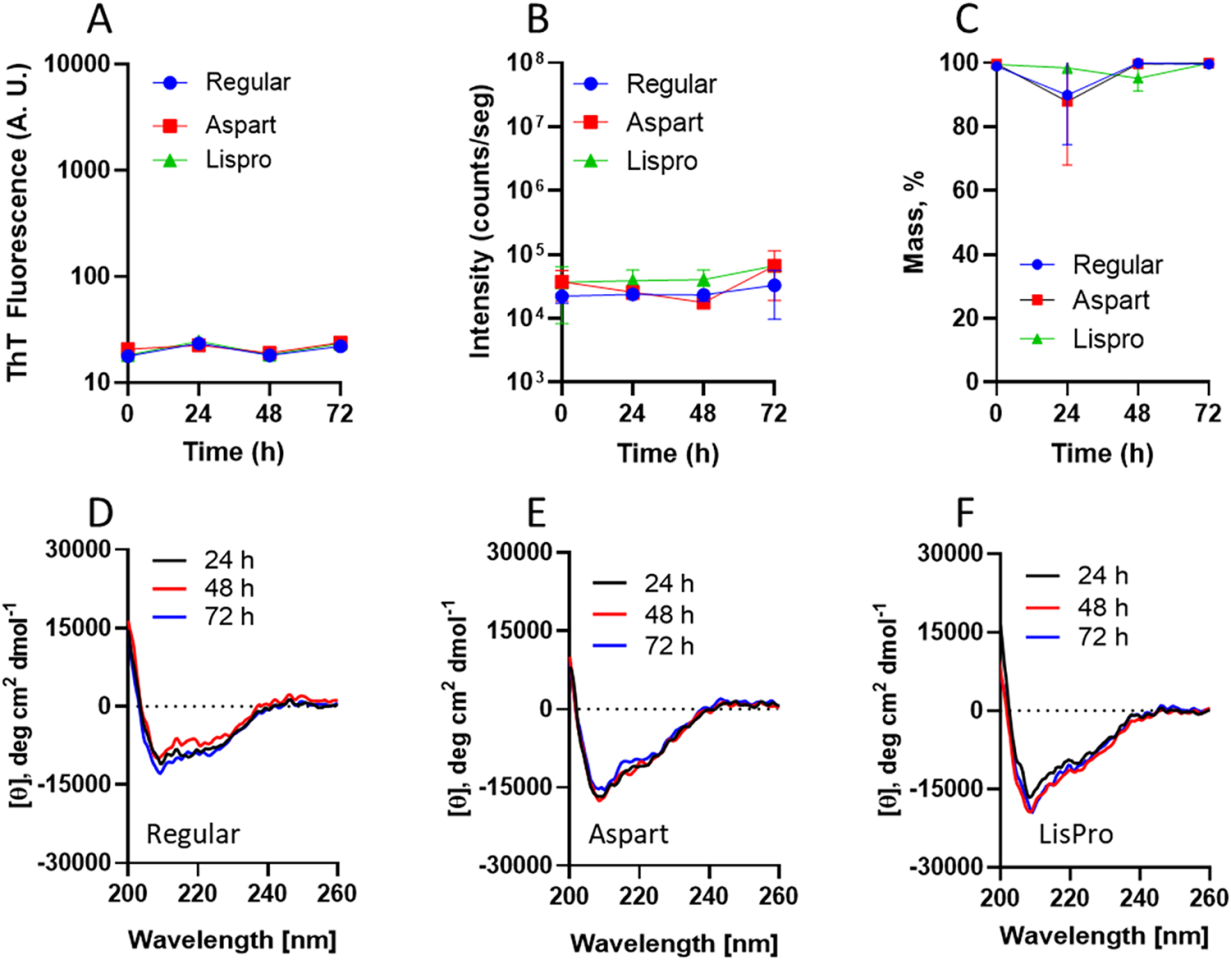
Physical stability of insulin infusions. Insulin (regular human, blue; Aspart, red; LisPro, green) (1 IU/mL) was incubated in IIP saline flasks at 25 °C and assayed for amyloid (by ThT fluorescence; A) and aggregates (by DLS, (B and C)) every 24 h for 3 days (*n* = 3; bars are standard deviation). (B) Total scattering intensity from DLS measurements. (C) Particle mass percentage up to 2.5 nm in hydrodynamic radius. At each time interval, insulin (regular human, (D); aspart (E); LisPro (F)) was evaluated for secondary-structure content by circular dichroism using saline as the buffer.

## 4 Discussion

Amyloid fibrillation is a general property of polypeptides, including insulin,^11,21^ that can occur under varying chemical and physical conditions.^22–29^ Biological products for therapeutic use can aggregate in both amorphous and amyloid form, and monitoring these events is important for safety and efficacy.

Formulated insulin (at 100 IU/mL in original vials) can aggregate into nonamyloid subvisible particles over its expiration date after first use at a similar rate when stored both at low (4 °C) or high (37 °C) temperatures,^18^ which was found to be only detectable by DLS. Here, we have shown that when human insulin is diluted to 1 IU/mL (34.5 μg/mL) in saline, under conditions similar to those used in clinical practice in hospital, no subvisible particles, amyloid material, or interconversion into β-sheet-rich structures is detectable up to 72 h of incubation at 25 °C (Figure 5), although it may occur within 24 h at 37 °C (Figure S3) In our study, DLS would be able to detect subvisible particles as low as 0.05 IU/mL (1.72 μg/mL), corresponding to 5% of the insulin concentration in the IIP, while insulin fibrillation was found to occur at concentrations as low as 0.1 IU/mL (3 μg/mL) (Figures S2 and S3). The presence of non-native insulin aggregates as subvisible particles at lower amounts cannot be ruled out.

Amyloid detection by ThT is well-established and adopted in the antemortem diagnosis of amyloid diseases from varying biological matrices, such as CSF, skin, nasal swabs, among others.^30–36^ ThT is also widely used in seed amplification assays for detecting amyloid material.^35,36^ After standardizing insulin amyloid formation in our analytical setups, we found that amyloid material was not detected in the insulin infusions within the detection limit. Smaller amounts of amyloid material could be present and detected if using a system designed for amyloid seed amplification assay of insulin, such as those designed for α-synuclein or prion,^35,37^ although it is currently not available for insulin.

Biosynthetic insulin is well-standardized internationally and shows high physicochemical and structural identity.^38,39^ Additionally, we have shown that two brands of human insulin exhibit similar biophysical properties during their shelf life after opening.^18^ In this context, the interpretation of the present data on human insulin might be extended to other brands. Nonetheless, further investigations are required to address the formation of subvisible particles over longer incubation period in saline at 1 IU/mL, in broader temperature range (e.g., 15–30 °C), in other diluents (Ringer, dextrose, although not in clinical practice), in other saline flasks made of different materials, and the interaction with IIP tubing, in order to characterize the physical compatibility of insulin and these variables and thus the best conditions and the limitations of the IIP.

Insulin hexamers are stabilized by phenolic compounds (phenol and meta-cresol), while monomers can be favored by destabilizing compounds such as niacinamide.^40^ Metals and chelating agents can also modulate the oligomer stability and propensity for amyloid fibrillation.^41,42^ The properties of the biocompatible insulin-stabilizing agent can be explored in order to gain further protection against amorphous and amyloid aggregation in insulin infusions. Furthermore, we propose that the current approach for detecting amyloid and subvisible particles should be considered for other biopharmaceuticals.

## 5 Conclusions

Insulin is prone to amorphous and amyloid aggregation under varying physical and chemical circumstances, including neutral saline and phosphate buffer. No significant amount of subvisible insulin particles, either amorphous or amyloid, β-sheet-rich structures, could be detected at a level of 0.05 IU/mL or higher by the highly sensitive dynamic light scattering in saline infusions of insulin at 1 IU/mL up to 72 h, suggesting the stability of this extemporaneous formulation in saline flasks for hospital usage. While Thioflavin T (ThT) is a reliable method for detecting amyloid material, no amyloid was detected in our insulin infusions within the detection limits of our current setup. However, further investigation of trace amounts of amyloid material might be performed by using amyloid seed amplification assays, which are not yet available for insulin. The methodologies for detecting HMWP, both amyloid and amorphous, and other subvisible particles should be considered for broader application to other biopharmaceuticals.

## Supplementary Material



## Data Availability

All raw data generated during the current study are available from the corresponding author on reasonable request.

## Notes

Hydrodynamic properties of native insulin by DLS (Table S1). HT data for circular dichroism (Figure S1); Insulin amyloid fibrillation by the *du Vigneaud* acid/heat method (Figure S2); Insulin fibrillation in saline (Figure S3); Insulin stability in saline (Figure S4); and Spectroscopic stability of insulin in saline (Figure S5) (PDF)

## Abbreviation

Aspart: ^B29^Asp-human insulin
cryoEM: cryoelectron microscopy
COC: cyclic olefin copolymer
DLS: dynamic light scattering
IIP: insulin infusion protocol
LisPro: B28Lys-B29Pro-human insulin
PBS: phosphate-buffered saline
PP: polypropylene
TEM: transmission electron microscopy
ThT: thioflavin T
USP: United States Pharmacopeia

## References

[1] Goldberg P. A., Siegel M. D., Sherwin R. S., Halickman J. I., Lee M., Bailey V. A., Lee S. L., Dziura J. D., Inzucchi S. E. (2004). Implementation
of a Safe and Effective Insulin Infusion Protocol in a Medical Intensive
Care Unit. Diabetes Care.

[2] Jacobi J., Bircher N., Krinsley J., Agus M., Braithwaite S. S., Deutschman C., Freire A. X., Geehan D., Kohl B., Nasraway S. A., Rigby M., Sands K., Schallom L., Taylor B., Umpierrez G., Mazuski J., Schunemann H. (2012). Guidelines
for the Use of an Insulin Infusion for the Management of Hyperglycemia
in Critically Ill Patients. Crit. Care Med..

[3] Shetty S., Inzucchi S. E., Goldberg P. A., Cooper D., Siegel M. D., Honiden S. (2012). Adapting To the New Consensus Guidelines for Managing
Hyperglycemia During Critical Illness: The Updated Yale Insulin Infusion
Protocol. Endocr. Pract..

[4] Van
den Berghe G., Wouters P., Weekers F., Verwaest C., Bruyninckx F., Schetz M., Vlasselaers D., Ferdinande P., Lauwers P., Bouillon R. (2001). Intensive Insulin Therapy
in Critically Ill Patients. N. Engl. J. Med..

[5] Greenwood B. C., Chesnick M. A., Szumita P. M., Belisle C., Cotugno M. (2012). Stability
of Regular Human Insulin Extemporaneously Prepared in 0.9% Sodium
Chloride in a Polyvinyl Chloride Bag. Hosp.
Pharm..

[6] Henry H., Gilliot S., Genay S., Barthélémy C., Décaudin B., Odou P. (2022). Stability of 1-Unit/mL Insulin Aspart
Solution in Cyclic Olefin Copolymer Vials and Polypropylene Syringes. Am. J. Health. Syst. Pharm..

[7] Blatherwick N. R., Bischoff F., Maxwell L. C., Berger J., Sahyun M. (1927). Studies on
insulin. J. Biol. Chem..

[8] Du
Vigneaud V., Geiling E. M. K., Eddy C. A. (1928). Studies on crystalline
insulin vi. further contributions to the question whether or not crystalline
insulin is an adsorption product. J. Pharmacol.
Exp. Ther..

[9] du
Vigneaud V., Sifferd R. H., Sealock R. R. (1933). The heat precipitation
of insulin. J. Biol. Chem..

[10] Waugh D. F. (1946). A Fibrous
Modification of Insulin. I. The Heat Precipitate of Insulin. J. Am. Chem. Soc..

[11] Brange, J. Galenics of Insulin; Springe: Berlin, Heidelberg, 1987 10.1007/978-3-662-02526-0.

[12] Dische F. E., Wernstedt C., Westermark G. T., Westermark P., Pepys M. B., Rennie J. A., Gilbey S. G., Watkins P. J. (1988). Insulin
as an Amyloid-Fibril Protein at Sites of Repeated Insulin Injections
in a Diabetic Patient. Diabetologia.

[13] Albert S. G., Obadiah J., Parseghian S. A., Yadira Hurley M., Mooradian A. D. (2007). Severe Insulin Resistance Associated
with Subcutaneous
Amyloid Deposition. Diabetes Res. Clin. Pract..

[14] da
Silva D. C., Lima L. M. T. R. (2018). Physico-Chemical Properties of Co-Formulated
Fast-Acting Insulin with Pramlintide. Int. J.
Pharm..

[15] Wang L., Hall C. E., Uchikawa E., Chen D., Choi E., Zhang X., Bai X. (2023). Structural Basis of
Insulin Fibrillation. Sci. Adv..

[16] Tarr B. D., Campbell R. K., Workman T. M. (1991). Stability and Sterility of Biosynthetic
Human Insulin Stored in Plastic Insulin Syringes for 28 Days. Am. J. Health. Syst. Pharm..

[17] Richter B., Bongaerts B., Metzendorf M.-I. (2023). Thermal Stability and Storage of
Human Insulin. Cochrane Database Syst. Rev..

[18] Silva-Jr H., Araújo T. S., da Silva Almeida M., Scapin S. M. N., Lima L. M. T. R. (2022). Formation
of Subvisible Particles in Commercial Insulin Formulations. Colloids Surf., B.

[19] Bradford M. M. (1976). A Rapid
and Sensitive Method for the Quantitation of Microgram Quantities
of Protein Utilizing the Principle of Protein-Dye Binding. Anal. Biochem..

[20] LeVine H. (1999). Quantification
of Beta-Sheet Amyloid Fibril Structures with Thioflavin T. Methods Enzymol..

[21] Fink A. L. (1998). Protein
Aggregation: Folding Aggregates, Inclusion Bodies and Amyloid. Fold. Des..

[22] Fändrich M., Fletcher M. A., Dobson C. M. (2001). Amyloid Fibrils
from Muscle Myoglobin. Nature.

[23] Fändrich M., Dobson C. M. (2002). The Behaviour
of Polyamino Acids Reveals an Inverse
Side Chain Effect in Amyloid Structure Formation. EMBO J..

[24] Pearce F. G., Mackintosh S. H., Gerrard J. A. (2007). Formation of Amyloid-like Fibrils
by Ovalbumin and Related Proteins under Conditions Relevant to Food
Processing. J. Agric. Food Chem..

[25] Palmieri L. C., Melo-Ferreira B., Braga C. A., Fontes G. N., Mattos L. J., Lima L. M. T. R. (2013). Stepwise
Oligomerization of Murine Amylin and Assembly
of Amyloid Fibrils. Biophys. Chem..

[26] da
Silva D. C., Fontes G. N., Erthal L. C. S., Lima L. M. T. R. (2016). Amyloidogenesis
of the Amylin Analogue Pramlintide. Biophys.
Chem..

[27] Sivalingam V., Prasanna N. L., Sharma N., Prasad A., Patel B. K. (2016). Wild-Type
Hen Egg White Lysozyme Aggregation in Vitro Can Form Self-Seeding
Amyloid Conformational Variants. Biophys. Chem..

[28] Lima L. M. T. R., Icart L. P. (2022). Amyloidogenicity
of Peptides Targeting Diabetes and
Obesity. Colloids Surf., B.

[29] Nirwal S., Bharathi V., Patel B. K. (2021). Amyloid-like
Aggregation of Bovine
Serum Albumin at Physiological Temperature Induced by Cross-Seeding
Effect of HEWL Amyloid Aggregates. Biophys.
Chem..

[30] Colby D. W., Zhang Q., Wang S., Groth D., Legname G., Riesner D., Prusiner S. B. (2007). Prion Detection
by an Amyloid Seeding
Assay. Proc. Natl. Acad. Sci. U. S. A..

[31] Atarashi R., Sano K., Satoh K., Nishida N. (2011). Real-Time Quaking-Induced
Conversion: A Highly Sensitive Assay for Prion Detection. Prion.

[32] Atarashi R., Satoh K., Sano K., Fuse T., Yamaguchi N., Ishibashi D., Matsubara T., Nakagaki T., Yamanaka H., Shirabe S., Yamada M., Mizusawa H., Kitamoto T., Klug G., McGlade A., Collins S. J., Nishida N. (2011). Ultrasensitive
Human Prion Detection in Cerebrospinal Fluid by Real-Time Quaking-Induced
Conversion. Nat. Med..

[33] Groveman B. R., Orrù C. D., Hughson A. G., Raymond L. D., Zanusso G., Ghetti B., Campbell K. J., Safar J., Galasko D., Caughey B. (2018). Rapid and
Ultra-Sensitive Quantitation of Disease-Associated
α-Synuclein Seeds in Brain and Cerebrospinal Fluid by αSyn
RT-QuIC. Acta Neuropathol. Commun..

[34] Wang Z., Becker K., Donadio V., Siedlak S., Yuan J., Rezaee M., Incensi A., Kuzkina A., Orrú C. D., Tatsuoka C., Liguori R., Gunzler S. A., Caughey B., Jimenez-Capdeville M. E., Zhu X., Doppler K., Cui L., Chen S. G., Ma J., Zou W.-Q. (2021). Skin α-Synuclein
Aggregation Seeding Activity as a Novel Biomarker for Parkinson Disease. JAMA Neurol..

[35] Concha-Marambio L., Pritzkow S., Shahnawaz M., Farris C. M., Soto C. (2023). Seed Amplification
Assay for the Detection of Pathologic Alpha-Synuclein Aggregates in
Cerebrospinal Fluid. Nat. Protoc..

[36] Duan S., Yang J., Cui Z., Li J., Zheng H., Zhao T., Yuan Y., Liu Y., Zhao L., Wang Y., Luo H., Xu Y. (2023). Seed Amplification
Assay of Nasal Swab Extracts for Accurate and Non-Invasive Molecular
Diagnosis of Neurodegenerative Diseases. Transl.
Neurodegener..

[37] Atarashi R., Wilham J. M., Christensen L., Hughson A. G., Moore R. A., Johnson L. M., Onwubiko H. A., Priola S. A., Caughey B. (2008). Simplified
Ultrasensitive Prion Detection by Recombinant PrP Conversion with
Shaking. Nat. Methods.

[38] Fávero-Retto M.
P., Palmieri L. C., Souza T. A. C. B., Almeida F. C. L., Lima L. M. T. R. (2013). Structural
Meta-Analysis of Regular Human Insulin in Pharmaceutical Formulations. Eur. J. Pharm. Biopharm..

[39] Fávero-Retto M.
P., Guerreiro L. H., Pessanha C. M., Palmieri L. C., Lima L. M. T. R. (2017). Polymorphic
Distribution of Proteins in Solution by Mass Spectrometry: The Analysis
of Insulin Analogues. Biologicals.

[40] Kildegaard J., Buckley S. T., Nielsen R. H., Povlsen G. K., Seested T., Ribel U., Olsen H. B., Ludvigsen S., Jeppesen C. B., Refsgaard H. H. F., Bendtsen K. M., Kristensen N. R., Hostrup S., Sturis J. (2019). Elucidating
the Mechanism of Absorption
of Fast-Acting Insulin Aspart: The Role of Niacinamide. Pharm. Res..

[41] Pounot K., Grime G. W., Longo A., Zamponi M., Noferini D., Cristiglio V., Seydel T., Garman E. F., Weik M., Foderà V., Schirò G. (2021). Zinc Determines
Dynamical Properties
and Aggregation Kinetics of Human Insulin. Biophys.
J..

[42] Mittal S., Prajapati K. P., Ansari M., Joshi K., Mishra N., Mahato O. P., Anand B. G., Kar K. (2024). Cu­(II) Specifically
Disassembles Insulin Amyloid Nanostructures via Direct Interaction
with Cross-β Fibrils. Nano Lett..

