# Supplementary material for: Physicochemical Stability of Insulin and Analogues in Saline Infusion: Screening for Amyloid and Amorphous High-Molecular-Weight Material
Source: ACS Omega. 2026 Mar 6;11(10):16481–8. doi: 10.1021/acsomega.5c12466 (PMC13000647; doi:10.1021/acsomega.5c12466)
Supplement: Supplementary file 1 [file ao5c12466_si_001.pdf]

SUPPORTING INFORMATION

Physico-chemical stability of insulin and analogues in saline infusion: screening for amyloid and amorphous high molecular weight material

João Gabriel da Cruz e Silva [1], Fernando de Sá Ribeiro[1], Luís Maurício T. R. Lima[1]*

[1] Laboratório de Biotecnologia Farmacêutica - pbiotech, Faculdade de Farmácia, Universidade Federal do Rio de Janeiro, Rio de Janeiro, RJ, 21941-902, Brazil.

***Corresponding author:**
Luís Maurício T. R. Lima - Laboratório de Biotecnologia Farmacêutica – pbiotech, Faculdade de Farmácia, Universidade Federal do Rio de Janeiro, Rio de Janeiro, RJ, 21941-902, Brazil.
Mauricio@farmacia.ufrj.br

João Gabriel da Cruz e Silva - joaosilva.jg91@gmail.com
Fernando de Sá Ribeiro – FernandoRibeiroBiomed@gmail.com
Luís Maurício T. R. Lima – LuisMauricioLima@gmail.com, Mauricio@farmacia.ufrj.br

Experimental

Amyloid fibrillation assay

Human insulin (wild-type, regular; Novolin R) and analogues (aspart, NovoRapid; LisPro, HumaLog) were purchased from local drugstores and kept refrigerated at all times until use. Insulin 100 U/mL corresponds to approximately 3.45 mg/mL. Composition of the insulin products is as described:

- **Human insulin**[1] - zinc 21 µg/mL, glycerol 16 mg/mL, m-cresol 3 mg/mL, W.F.I., HCl/NaOH to pH of approximately 7.0 – 7.8.
- **LisPro**[2] (^{B28}Lys^{B29}Pro) - 19.7 µg zinc ion, glycerol 16 mg/mL, m-cresol 3.15 mg/mL, trace amounts of phenol, Na_2HPO_4 1.0 mg/mL, W.F.I., HCl/NaOH to pH of approximately 7.0 to 7.8
- **Aspart**[3] (^{B29}Asp) - 19.6 µg/mL zinc, 16 mg/mL glycerol, 1.72 mg/mL m-cresol, 1.50 mg/mL phenol, 1.25 mg/mL $Na_2HPO_4.2H_2O$, 0.58 mg/mL NaCl, W.F.I. HCl/NaOH to pH of approximately 7.2-7.6

Insulin fibrillation assay was performed in multiwell plate and monitored by Thioflavin T as reported [14,20]. Insulin at varying dilutions in saline or HCl 0.1 M as indicated, in the presence of 30 µM ThT, incubated in 96-well, polystyrene flat bottom plate (Cralplast, Cat # 655111T or Kartell, Cat # 2620), and sealed with a transparent film (Crystal Clear Invisible Tape, Duck Brand, USA). Measurements were performed in a fluorescence spectrometer (FluorOmega, BMG; pbiotech, FF, UFRJ), with excitation with filter 440 nm and emission with filter 482 nm, gain 700, top read, with readings each 5 min preceded by 10 second double-orbital shaking (300 rpm) at the indicated temperature. Unbounded ThT fluorescence was not subtracted from samples since: i) the fluorescence of the free ThT is stable over time (**Fig. S1**), ii) free ThT fluorescence is below the amyloid fibril-bound ThT fluorescence iii) the amount of free ThT is not constant, decreasing over time due to its binding to fibrillated protein. Assays were performed in quadruplicate.

Stability assay in saline

Sterile saline flasks (0.9 % NaCl in water for injection; Ever Care, ADV-Farma, Brazil; High Density Polyethylene) were kept at room temperature (25 °C $\pm$ 2 °C) under mild rocking (8 oscillations/minute), and aliquots were withdrawn with sterile syringes for immediate measurements in a Genesys 10S UV/VIS spectrophotometer (Thermo Sci, USA), between 200 and 400 nm, with 0.5 nm step resolution, in a 10.0 mm quartz cuvette, using saline as blank. Assays were performed in triplicate.

[1] Novolin R, https://go.drugbank.com/drugs/DB00030, https://www.accessdata.fda.gov/drugsatfda_docs/label/2019/019938s079lbl.pdf
[2] Humalog, https://go.drugbank.com/drugs/DB00046, https://www.accessdata.fda.gov/drugsatfda_docs/label/2023/020563s202,021017s146,021018s132,205747s028lbl.pdf
[3] Novorapid, https://go.drugbank.com/drugs/DB01306, https://www.accessdata.fda.gov/drugsatfda_docs/label/2019/020986s090s091lbl.pdf

Dynamic Light Scattering (DLS)

DLS measurements were performed on a DynaPro (Wyatt, USA; LaBiME, FF, UFRJ) at room temperature (25 ^{o}C + 2 ^{o}C) using a 45 µL quartz cuvette (Wyatt; Cat #WNQC-45-00). For each sample, 10 accumulations were measured. Assays were performed in triplicate.

Results

Table S1. Hydrodynamic properties of native insulin by DLS.
Numbers are mean and standard deviation (n=3)

Insulin variant	Hydrodynamic Radius (nm)	Standard Deviation (nm)
**Human, wild-type**	2.00	0.46
**Aspart**	3.03	1.78
**LisPro**	1.03	0.22

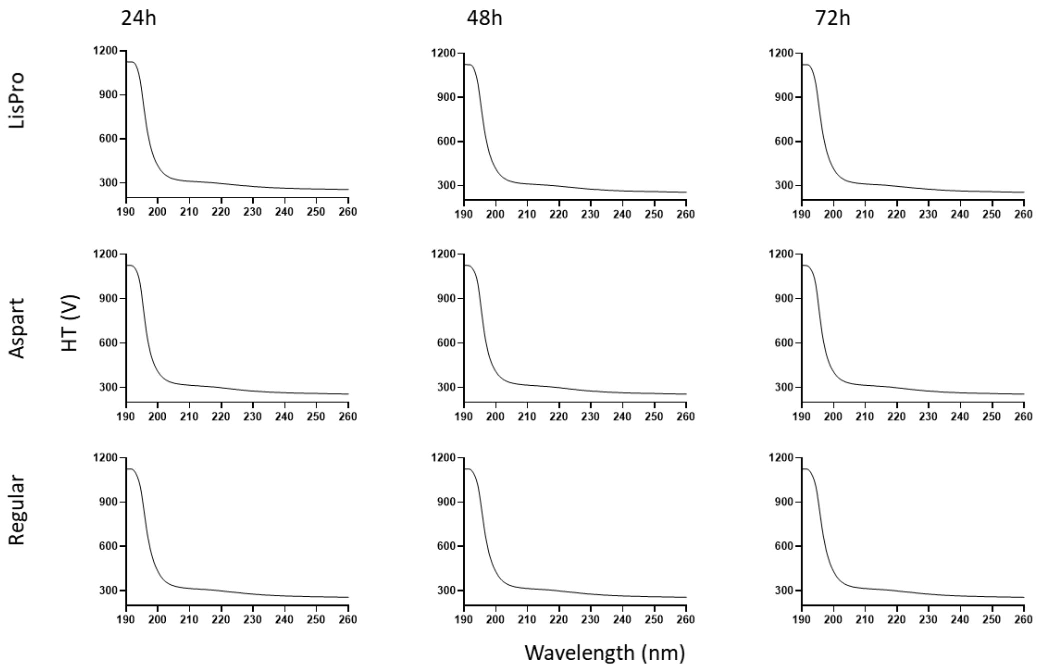

Figure S1. HT data for circular dichroism.

The HT spectra (from data in **Fig. 5**) of insulin (1-I.U./mL in saline, 25 ºC) show an increase in the far-UV region, at wavelengths below 200 nm, due to interference of the high salt concentration in the saline.

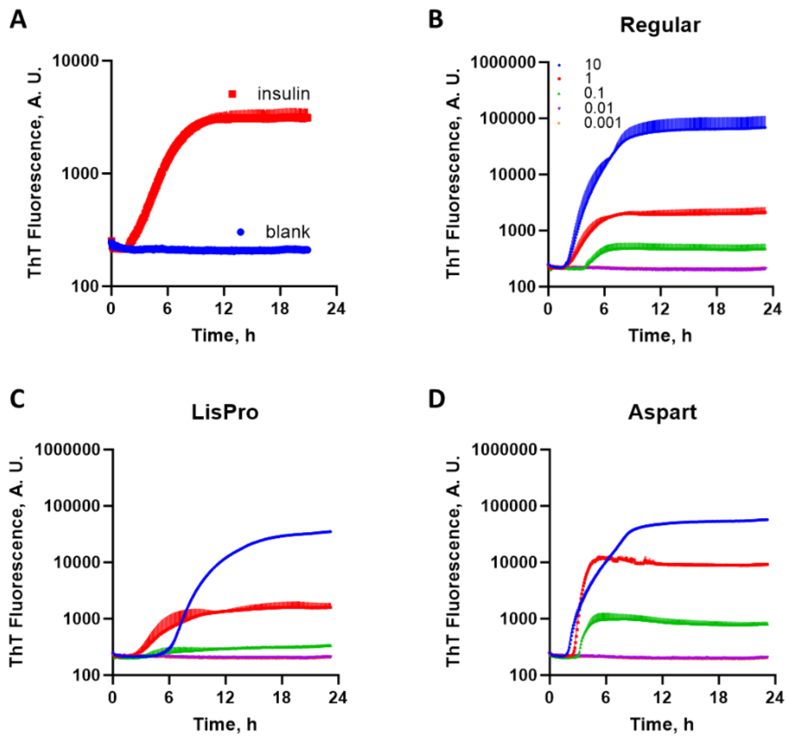

Figure S2. Insulin amyloid fibrillation by the *du Vigneaud* acid/heat method.

A) Insulin was incubated at 1-I.U./mL in 100 mM HCl in the presence of 30 µM ThT at 45 ^{o}C, and the formation of amyloid material was monitored by the increase in fluorescence, which did not occur in the control samples (blank) in the absence of protein. Insulin amyloid fibrillation is concentration dependent, not detected at 0.01-I.U./mL or below for (regular) human wild-type (**B**), LisPro (**C**) and Aspart (**D**), with colors corresponding to concentrations as in (**B**). Symbols are mean and standard deviation (n=4).

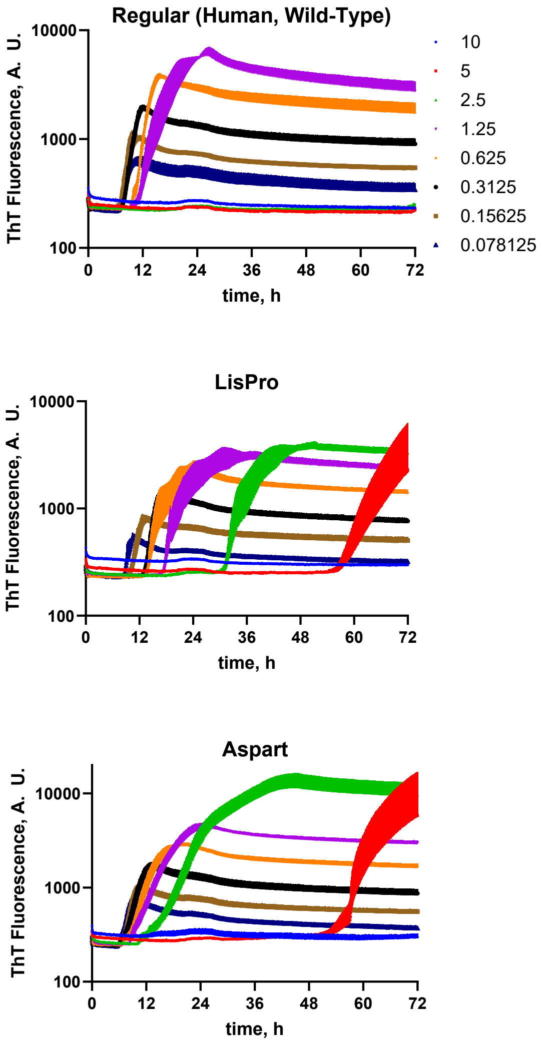

Figure S3. Insulin fibrillation in saline.

Insulin (human wild-type, LisPro and Aspart) at varying concentrations (from 10 IU/mL to 0.078 IU/mL in 2x dilution series) was incubated in saline 0.9 % with 30 μM ThT at 37 °C and monitored for amyloid formation as indicated by the increase in ThT fluorescence. Symbols are mean and bars are standard error of the mean (n=4). Notice fibrillation occurring from 5 I.U./mL to 0.078 I.U./mL

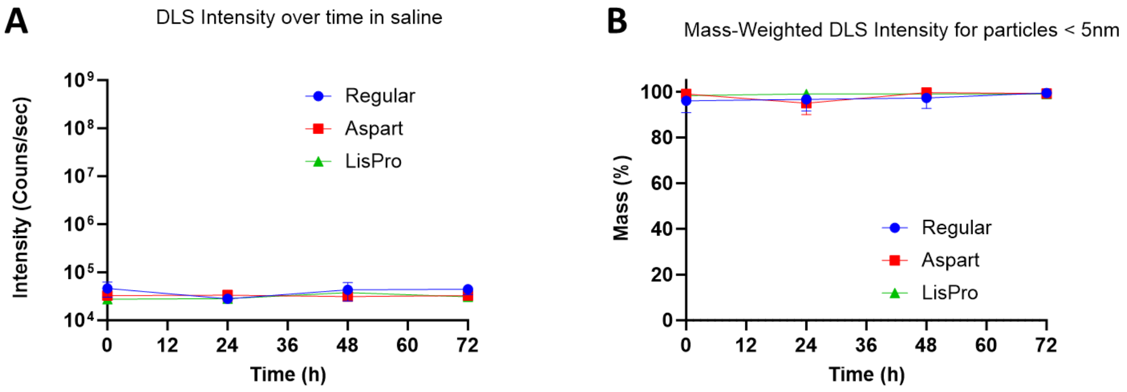

Figure S4. Insulin stability in saline.

Insulin (regular human, wild-type; LisPro; Aspart; 1 I.U./mL) was incubated at 25 °C in saline (100 mL) for up to 72h and evaluated for stability by the formation of high molecular weight particles (HMWP) by (**A**) the changes (eventual increase) in total DLS intensity and (**B**) changes (eventual decrease) in the mass contribution of particles less than 5 nm in Rh, corresponding to the native insulin as shown in **Table S1**. Values are mean and standard deviation (n=3).

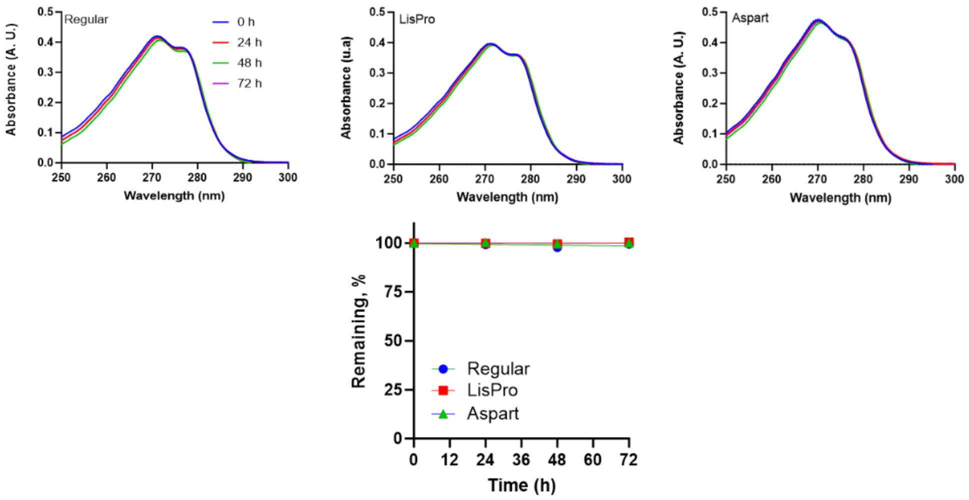

Figure S5. Spectroscopic stability of insulin in saline

UV–Vis absorbance spectra of human insulin and insulin analogues (Lispro and Aspart) in saline (1 U/mL), at 25 ^{o}C, monitored over 72 h. Spectra were recorded at 24 h intervals under quiescent conditions at 25 °C. Data is mean and standard deviation (n=3). Kinetic data show no change (A_{272nm}) up to 72 h, with slope for first-order linear regression not different from zero ($p>0.5$) Human Insulin (UNIPROT P01308) would decrease by about A_{280nm} = 0.073 (according to Expasy ProtParam) for 1 I.U./mL (34.5 µg/mL) in case of loss of protein from solution, or increase the baseline (e.g., over 300 nm) due to transmittance attenuation by turbidity (not shown). Values are mean and standard deviation (n=3).
